# Insect Herbivory-Elicited GABA Accumulation in Plants is a Wound-Induced, Direct, Systemic, and Jasmonate-Independent Defense Response

**DOI:** 10.3389/fpls.2015.01128

**Published:** 2015-12-22

**Authors:** Sandra S. Scholz, Michael Reichelt, Dereje W. Mekonnen, Frank Ludewig, Axel Mithöfer

**Affiliations:** ^1^Department of Bioorganic Chemistry, Max Planck Institute for Chemical EcologyJena, Germany; ^2^Department of Biochemistry, Max Planck Institute for Chemical EcologyJena, Germany; ^3^Department Botany II, Cologne Biocenter, University of CologneCologne, Germany; ^4^Division of Biochemistry, Department of Biology, University of Erlangen-NurembergErlangen, Germany

**Keywords:** γ-aminobutyric acid, *Arabidopsis thaliana*, herbivory, *Spodoptera littoralis*, MecWorm, jasmonate, wounding, plant defense

## Abstract

The non-proteinogenic amino acid γ-aminobutyric acid (GABA) is present in all organisms analyzed so far. In invertebrates GABA acts as a neurotransmitter; in plants different functions are under discussion. Among others, its involvement in abiotic stress reactions and as a defensive compound against feeding insects is suggested. GABA is synthesized from glutamate by glutamate decarboxylases and degraded by GABA-transaminases. Here, in *Arabidopsis thaliana, gad1/2* double mutants showing reduced GABA concentrations as well as GABA-enriched triple mutants (*gad1/2 x pop2-5*) were generated and employed for a systematic study of GABA induction, accumulation and related effects in *Arabidopsis* leaves upon herbivory. The results demonstrate that GABA accumulation is stimulated by insect feeding-like wounding by a robotic caterpillar, MecWorm, as well as by real insect (*Spodoptera littoralis*) herbivory. Higher GABA levels in both plant tissue and artificial dietary supplements in turn affect the performance of feeding larvae. GABA enrichment occurs not only in the challenged but also in adjacent leaf. This induced response is neither dependent on herbivore defense-related phytohormones, jasmonates, nor is jasmonate induction dependent on the presence of GABA. Thus, in *Arabidopsis* the rapid accumulation of GABA very likely represents a general, direct and systemic defense reaction against insect herbivores.

## Introduction

The four carbon non-proteinogenic amino acid γ-aminobutyric acid (GABA) is widespread in animals, plants, and microorganisms. GABA is mainly synthesized by decarboxylation of L-glutamate in the cytosol (Breitkreuz and Shelp, 1995). The reaction is carried out by glutamate decarboxylases (GAD, EC 4.1.1.15). In *Arabidopsis*, five genes encoding GADs exist and show organ specificity. Whereas *GAD2* transcripts are detectable in all organs, *GAD5* transcripts are detected in male gametes and GAD1 expresses mainly in roots (Turano and Fang, 1998; Zik et al., 1998; Hruz et al., 2008). It is suggested that GAD activity is regulated by pH and Ca^2+^/calmodulin (Carroll et al., 1994; Snedden et al., 1995; Bown et al., 2006). At neutral pH, GAD activity depends on Ca^2+^/calmodulin; however, any acidification of the cytosol, for example by wounding-mediated disruption of the vacuole, can stimulate GAD activity independent on Ca^2+^/calmodulin. In addition, GABA can also be produced from polyamines (Shelp et al., 2012a). Catabolism of GABA is localized in the mitochondrial matrix. A GABP (GABA permease) transporter encoded by a single copy gene in *Arabidopsis* has been described to import GABA into mitochondria (Michaeli et al., 2011). However, the lack of a prominent phenotype of the loss-of-function *gabp* mutant argues for the presence of other transporters capable of importing GABA into mitochondria. Once in the mitochondrial matrix, a transaminase reaction catalyzed by a GABA transaminase (GABA-T) moves the amino group of GABA onto pyruvate yielding alanine and in parallel succinic semialdehyde (SSA) (Clark et al., 2009). In *Arabidopsis*, GABA-T is also encoded by a single copy gene. Disruption of the *GABA-T* gene leads to strong GABA accumulation. In the vegetative growth phase, no prominent phenotype of *gaba-t* mutants can be observed, however, fertility is decreased in the mutant due to impaired pollen tube growth (Palanivelu et al., 2003; Renault et al., 2011; Yu et al., 2014). SSA is either exported from mitochondria by a yet unknown transporter and further metabolized (Breitkreuz et al., 2003), or is oxidized to succinate, a tricarboxylic acid (TCA) cycle intermediate, by succinic semialdehyde dehydrogenase (SSADH). Disruption of the single copy *SSADH* gene leads to a severe phenotype (Bouché et al., 2003; Ludewig et al., 2008). It has been shown that accumulation of SSA is causative for the production of leaf necrosis and impaired growth of *ssadh* mutants due to the production of reactive oxygen species (Fait et al., 2005).

So far, GABA has been found in all plant species investigated (Shelp et al., 2009). It has been mostly considered as a metabolite involved in the control of C/N balance and in anaplerotic alimentation of the Krebs cycle (Fait et al., 2008). However, the function of GABA in plants is far from being resolved. Several findings started a discussion about GABA functioning as a signaling compound in plant growth and development (Bouché and Fromm, 2004). For example, in *Arabidopsis thaliana* it was shown that pollen tube-growth in pistils as well as hypocotyl- and root-growth depend on controlled low GABA levels (~1 nmol g^−1^ DW) (Palanivelu et al., 2003; Renault et al., 2011). Again in *Arabidopsis*, low GABA levels are important and a prerequisite for *E*-2-hexenal-induced root growth inhibition (Mirabella et al., 2008). In all these cases it was demonstrated that mutations in the same gene (*GABA-T*) encoding a γ-amino butyric acid transaminase, caused enhanced GABA levels in the resulting *pop2* and *her1* mutant plants (Palanivelu et al., 2003; Mirabella et al., 2008; Renault et al., 2011). The increased endogenous concentration of GABA seems to be the reason for impaired cell elongation in the mutants and the corresponding phenotypes (Renault et al., 2011). Other studies demonstrated that GABA is involved in the differentiation of the vascular system in pine (*Pinus pinaster*) seedlings (Molina-Rueda et al., 2015). Shelp et al. (2006) also suggested that GABA might be involved in the communication between plants and other organisms such as fungi, bacteria, and certain invertebrates (Shelp et al., 2006).

For many years it has been known that GABA accumulates in plants upon various abiotic stress challenges such as mechanical stimulation and tissue damage, salt and cold stress (Wallace et al., 1984; Ramputh and Bown, 1996; Shelp et al., 1999; Kinnersley and Turano, 2000; Renault et al., 2010). Recently, it was shown that GABA negatively regulates anion flux through plant aluminum-activated malate transporter (ALMT) and probably mediates its physiological effects via ALMT (Ramesh et al., 2015). GABA is also suggested to be involved in plant defense against herbivorous insects (Bown et al., 2006; Huang et al., 2011; Mithöfer and Boland, 2012). This hypothesis is based on several facts and observations: (i) GABA is known as an inhibitory neuromuscular transmitter acting at GABA-gated chloride channels in invertebrates, including insects, where it could affect normal development when ingested by feeding (Bown et al., 2006; Shelp et al., 2009). Thus, the presence of GABA might deter feeding of herbivorous insect as shown for *Choristoneura rosaceana* (oblique-banded leafroller) larvae raised on a synthetic diet (Ramputh and Bown, 1996). (ii) Leaf tissues of soybean (*Glycine max*) and tobacco (*Nicotiana tabacum*) that were only slightly wounded by crawling insect species (*C. rosaceana* and the tobacco budworm, *Heliothis virescens*, respectively) showed a GABA accumulation up to 4- to 12-fold within 5–10 min (Bown et al., 2002). (iii) Transgenic *N. tabacum* plants with elevated GABA levels due to constitutive transgenic expression of a GAD enzyme were more resistant to both *H. virescens* larvae and *Meloidogyne hapla*, the root-knot nematode (MacGregor et al., 2003; McLean et al., 2003; Bown et al., 2006).

In 2006, Bown and colleagues postulated in an opinion article “…that wounding stimulates gamma-aminobutyrate (GABA) accumulation in plants, which in turn deters herbivory by invertebrate pests” (Bown et al., 2006). Nearly a decade later, there is still a lack of experimental proof concerning the type of herbivory-related stimulus that is necessary, and the amount of which is sufficient, to induce GABA accumulation in plant leaves and whether this GABA contributes to the plant's defense. Here, we address these questions systematically. Moreover, many herbivory- or wounding-related defense responses in plants are strongly dependent on and mediated by jasmonates, fatty acid-derived phytohormones (Wasternack, 2007; Mithöfer et al., 2009). Thus, we also examined whether the induced defense of GABA accumulation is a jasmonate-regulated process.

## Materials and methods

### Plant and insect material, growth and plant treatment

Four to five week old *Arabidopsis thaliana* plants (wild-type: ecotype Col-0; mutants: *gad1/2, gad1/2 x pop2-5, jar1*) were used for all experiments. All plants were grown in 10 cm round pots as described elsewhere (Vadassery et al., 2012). For this seeds were sown and stratified for 2 days at 4°C, afterwards plants were kept at 40% humidity and 23°C. The growth conditions were adjusted to 10-h-light/14-h-dark photoperiod with a light intensity of 150 μmol m^2^ s^−1^. All plants used for one experiment germinated at the same day and were kept in the same growth chamber. When different plant lines were used at the same time they were kept separated from each other to avoid any kind of contact and placed randomized in the experimental setup. Larvae of generalist herbivore *Spodoptera littoralis* were hatched from eggs and reared on an agar-based optimal diet at 23–25°C with 8 h light/ 16 h dark cycles (Bergomaz and Boppre, 1986). For 7 days feeding assay, 1st instar larvae were used (they were kept in light for 3 days after hatching). The larvae were pre-weighed to ensure equal starting conditions for all experiments. For short term feeding assays (3 h), 4th instar *S. littoralis* larvae which were starved overnight prior to plant feeding were used. For coronalon (structural mimic of JA-Ile) treatment the randomly placed plants were sprayed with 1 ml of a 50 μM solution (0.1% ethanol, equivalent to 50 nmol) or solvent control and incubated with a cover to prevent evaporation. The coronalon used was synthesized according to Nakamura et al. (2014). Mechanical wounding was performed as described earlier using MecWorm (Scholz et al., 2014). To discriminate between a local and a systemic accumulation of GABA, leaves of plants were counted according to Farmer et al. (2013). Leaf number 8 was treated with MecWorm for 1 h; leaf 8 (local) as well as different systemic leaves (5, 9, and 11) were harvested. In other experiments fully developed leaves 9, 10, or 11 were used.

### *S. littoralis* growth inhibition assay with GABA

To determine growth effects of GABA on *S. littoralis*, 2nd instar larvae were reared on an artificial diet (see above) containing defined amounts of GABA (solved in water). A 0.5 M GABA stock solution was diluted several times; 100 μL of each were dropped on weighed pieces (1 g) of the artificial diet to give final concentrations of 0, 0.01, 0.1, 0.5, and 1 μmol GABA (g diet)^−1^. All insects were kept separated. The food was renewed every second day while the GABA concentration was maintained. The larval weight was determined before the experiment was started (day 0) and after 7 days of feeding. To calculate the growth inhibition, the measured increase in weight at different GABA concentrations was correlated with the control (no GABA, set to 100%).

### Generation of single, double, and triple mutants

The seeds of the single mutants *gad1* (At5g17330; SALK_017810), *gad2* (At1g65960; GK_474E05) and *pop2-5* (At3g22200; GK_157D10) were obtained from the respective stock centers. SALK lines were purchased from NASC (Nottingham *Arabidopsis* Stock Centre) and GABI-Kat (GK) lines were purchased from GABI-Kat directly (Alonso et al., 2003; Kleinboelting et al., 2012). F2 plants were screened for homozygousity by genotyping. For that, genomic DNA extraction from the individual plants was carried out as follows. Leaf samples were collected in 1.5 mL Eppendorf tubes containing 2–3 glass beads of 2 mm in size and snap-frozen in liquid nitrogen. The samples were crushed to powder using a tissue lyzer (Qiagen, Cat No 85220) for 3 min at a frequency of 20 s^−1^. Then, 200 μL of extraction buffer (0.2 M Tris HCl pH 7.5, 25 mM EDTA, 0.5 % SDS and 250 mM NaCl) were added and homogenized. The mixture was spun down for 1 min at 14,000 rpm, and 150 μL of the supernatant was transferred into new tubes. Next, an equal volume of 100 % isopropanol was added, mixed and incubated at room temperature for 5 min. Finally, the mixture was spun down at 14,000 rpm for 5 min, and the pellet was dissolved in 100 μL ddH_2_O. PCR analysis was performed using 2 μL of the DNA extract. For the generation of the *gad1/2* double mutant, the respective single mutants were crossed by emasculating the mother plant followed by pollination with the pollen from the male parent. For the isolation of homozygous double mutants, a similar procedure was followed as for the single mutants. The triple mutant was generated by crossing the homozygous *gad1/2* double mutant with the homozygous *pop2-5* mutant. The screening procedure was carried out as described above.

### RNA extraction, cDNA synthesis, and RT-PCRs for mutant characterization

Leaf samples (~100–200 mg) were collected from *Arabidopsis* plants and snap-frozen in liquid nitrogen. RNA extraction was carried out as described before with minor modifications (Logemann et al., 1987). Briefly, frozen tissue was crushed to powder using a pre-cooled electrical drill machine. Immediately, 1 mL of Z6 buffer (8 M guanidinium hydrochloride, 20 mM MES, 20 mM EDTA, pH 7.0) containing 0.7% (v/v) β-mercaptoethanol was added and homogenized by vortexing. Next, 500 μL PCI (phenol: chloroform: isoamylalcohol 25:24:1) were added and mixed by inverting the tube 10–15 times. After incubation for 3 min at room temperature, samples were spun down for 10 min at 4°C with 14,000 rpm. The aqueous phase (700 μL) was transferred to a new tube and 1/20 volumes acetic acid (1 M) and 0.7 volumes ethanol (100%) was added, mixed and incubated at room temperature for 10 min. The mix was spun down with 14,000 rpm for 10 min at 4°C. The pellet was then washed first with 500 μL of sodium acetate pH 5.0 followed by a second wash with 500 μL 70% ethanol. Finally, the pellet was air-dried and dissolved in 100 μL of RNase-free distilled water. Prior to cDNA synthesis the total RNA was treated with DNase (Promega) for 1 h at 37°C. The concentration of RNA was quantified using a NanoDrop (NanoDrop 1000 V.3.8), and the integrity of the RNA was verified on a 1% agarose gel. The cDNA was synthesized from 1.5 μg of total RNA in 20 μL of total reaction mixture according to the manufacturer's protocol (Bioscript). The synthesized cDNA was diluted three times and the expression of the target genes was analyzed using qRT-PCR. Used primers are listed in Table S1. As housekeeping gene *RPS18* was used according to Vadassery et al. (2012). The primers discriminating between the *GAD* paralogs have previously been reported except for *GAD5* (Renault et al., 2010).

### Quantification of phytohormones

For quantification of phytohormones 250 mg of sample were harvested and immediately frozen in liquid nitrogen and weighed. The extraction procedure and determination of JA and JA-Ile was carried out as described before (Vadassery et al., 2012). 60 ng of 9,10-D_2_-9,10-dihydrojasmonic acid, 60 ng of D_4_-salicylic acid, 60 ng of D_6_-ABA (Santa Cruz Biotechnology), and 15 ng of JA-[^13^C_6_]Ile conjugate were used as internal standards per sample. JA-[^13^C_6_]Ile conjugate was synthesized as described before (Kramell et al., 1988).

### Quantification of γ–aminobutyric acid (GABA)

Approximately 250 mg of fresh leaves were frozen in liquid nitrogen and weighed. The γ–aminobutyric acid (GABA) was extracted with 2 mL of methanol and the resulting extract was diluted in a ratio of 1:20 (v:v) in water containing the U-^13^C, ^15^N labeled amino acid mix (algal amino acids ^13^C, ^15^N, Isotec, Miamisburg, USA, at a concentration of 10 μg of the mix per ml). GABA in the diluted extracts was directly analyzed by LC-MS/MS. Chromatography was performed on an Agilent 1200 HPLC system (Agilent Technologies, Böblingen, Germany). Separation was achieved on a Zorbax Eclipse XDB-C18 column (50 × 4.6 mm, 1.8 μm, Agilent Technologies). Formic acid (0.05%) in water and acetonitrile were employed as mobile phases A and B, respectively. The elution profile was: 0–1 min, 3% B in A; 1–2.7 min, 3–100% B in A; 2.7–3 min 100% B and 3.1–6 min 3% B in A. The mobile phase flow rate was 1.1 mL/min. The column temperature was maintained at 25°C. The liquid chromatography was coupled to an API 5000 tandem mass spectrometer (Applied Biosystems, Darmstadt, Germany) equipped with a Turbospray ion source operated in positive ionization mode. The instrument parameters were optimized by infusion experiments with pure standards. The ion spray voltage was maintained at 5500 eV. The turbo gas temperature was set at 700°C. Nebulizing gas was set at 70 psi, curtain gas at 35 psi, heating gas at 70 psi and collision gas at 2 psi. Multiple reaction monitoring (MRM) was used to monitor analyte parent ion → product ion: GABA (*m/z* 104.1 → 87.1; DP 51, CE 17), U-^13^C, ^15^N-Ala (*m/z* 94.1 → 47.1; DP 51, CE 17). Both Q1 and Q3 quadrupoles were maintained at unit resolution. Analyst 1.5 software (Applied Biosystems) was used for data acquisition and processing. GABA in the sample was quantified using U-^13^C, ^15^N-Ala applying a response factor of 1.0.

## Results and discussion

### Two of five gad genes are mainly expressed in shoots and roots

**Figure 1:**
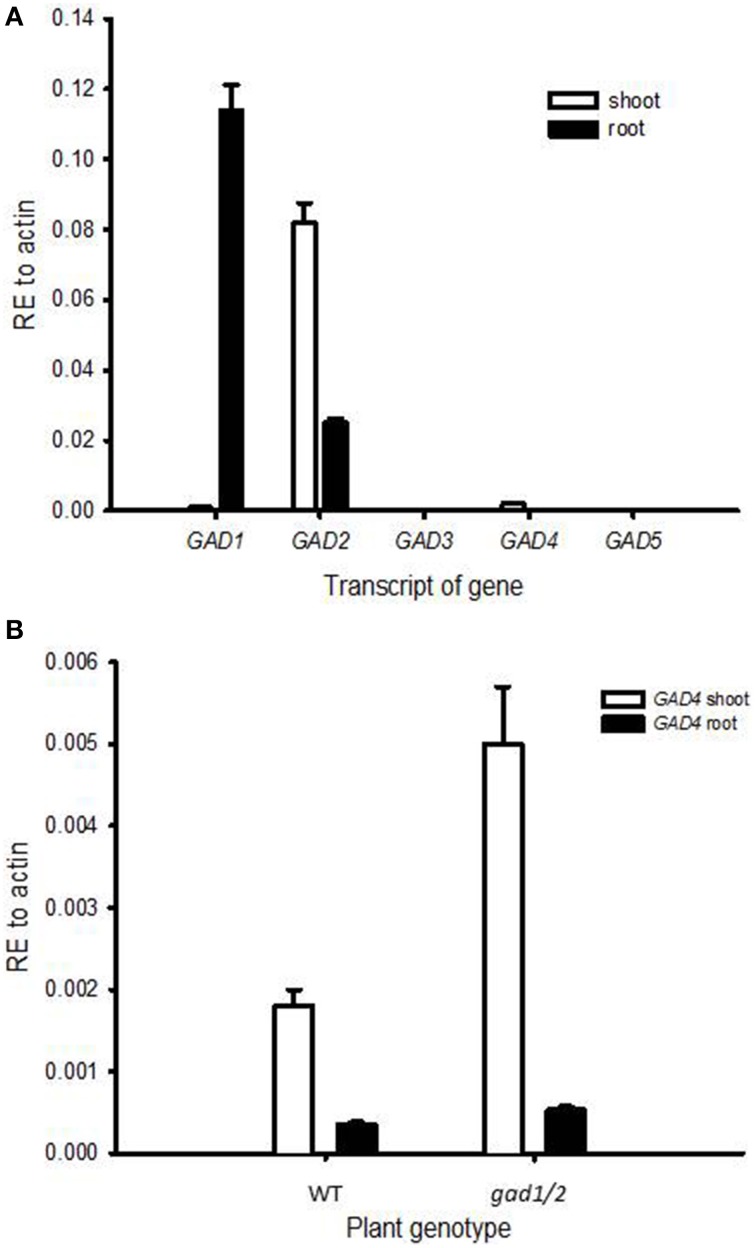
**Expression analysis of ***GAD*** genes in ***Arabidopsis*** shoots and roots**. Plants of 5-week-old wild-type **(A)** and 4-week-old wild-type and *gad1/2* mutant **(B)** were used. *GAD3* and *GAD5* transcripts were not detectable. Values are means of three biological replicates. Error bars represent the standard error of means; RE, relative expression.

In *Arabidopsis thaliana*, five *GAD* genes have been identified (Shelp et al., 1999). Here, we analyzed the relative expression of all five *GAD* paralogs in shoots and roots of wild-type plants. *GAD1* transcripts were mainly detected in roots (Figure 1A) and *GAD2* transcripts were abundantly detected in shoots and in considerable amounts in roots (Figure 1A), observations in line with previous findings (Turano and Fang, 1998; Zik et al., 1998). *GAD4*, on the other hand, exhibited a weak expression in shoots and an even weaker expression in roots (Figure 1). *GAD4* expression was also detected in flowers and siliques (Figure S1). The transcripts of *GAD3* and *GAD5* were neither detectable in shoots nor in roots. However, the transcript of *GAD3* could be detected in young siliques (Figure S1), and *GAD5* transcripts were detected in flowers (Figure S1). Indeed, strong expression of *GAD5* in gametes of *Arabidopsis thaliana* has been reported in publically available expression resources (http://www.bar.utoronto.ca/ and https://genevestigator.com/gv/, Winter et al., 2007; Hruz et al., 2008; Shelp et al., 2012b).

### A *gad1/2* double mutant contains low GABA amounts in shoots and roots

Next, we asked whether a simultaneous knock out of *GAD1* and *GAD2* would lead to major changes in the GABA pools of shoots and roots. To test that, we generated a *gad1/2* double mutant by crossing single *gad1* and *gad2* T-DNA insertion mutants (Figures S2A,B) and confirmed the absence of full-length transcripts (Figure S2C). However, a truncated *GAD2* transcript that consisted of exon 1, exon 2, and a part of exon 6, which is unable to encode a functional GAD, could be detected (Figures S2C,D). GADs belong to the pyridoxal phosphate-dependent aspartate aminotransferase super-family of proteins (Marchler-Bauer et al., 2011). Co-factor binding and catalytically active residues are encoded by bases located in exons 3, 4, and 5 of the native transcript. However, in the truncated version of the *GAD2* transcript, those exons were absent, and hence the protein very unlikely retains any decarboxylase activity. Furthermore, a premature stop codon has been detected close to the junction between the 2nd and the 6th exon to further shorten the unlikely functional protein (Figure S2D).

**Figure 2:**
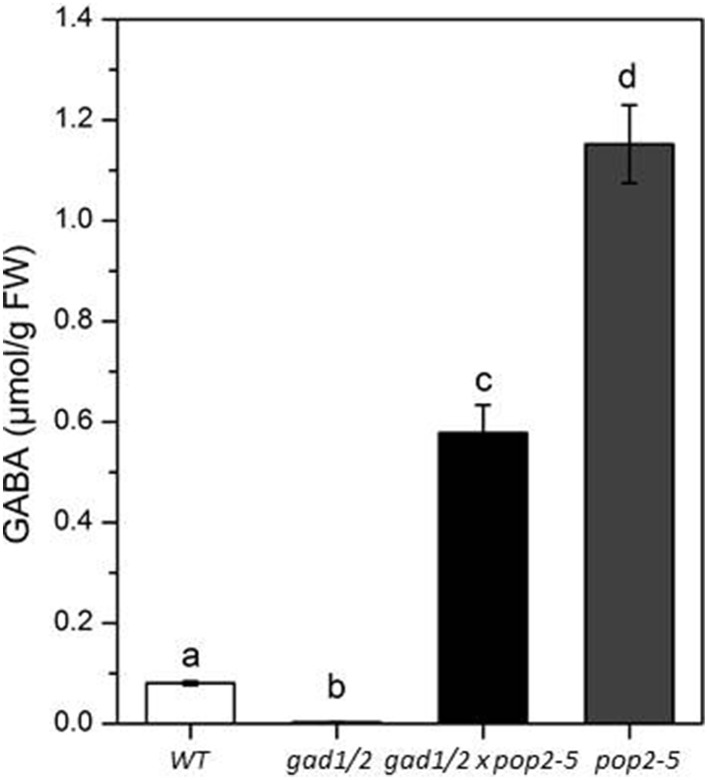
**GABA amounts of 4-week-old wild-type (WT), ***gad1/2***, ***gad1/2 x pop2-5***, and ***pop2-5*** plants**. All plants were grown under greenhouse conditions and GABA was quantified in leafs. Values are means of eight independent plants. Error bars represent the standard error of means. Statistically significant differences between WT and GABA mutant plants were analyzed by One-way ANOVA (*p* < 0.05, SNK-test) and are indicated by different letters.

The GABA content of the *gad1/2* double mutant was only 5% of that in the wild type (Figure 2). Despite reports indicating the possible synthesis of GABA from the degradation of polyamines (Bouchereau et al., 1999; Fait et al., 2008; Shelp et al., 2012a), GABA in *A. thaliana* seems to be mainly produced from the decarboxylation of glutamate by the activity of GADs. However, GABA concentration of *gad1/2* double mutants were not below the detection limit, either because of the above mentioned degradation of polyamines fueling GABA synthesis or because of low expression of *GAD4* (Figure 1). To examine whether an additional compensatory expression of *GAD* paralogs in *gad1/2* mutants occurred, the transcript levels of *GAD4* were analyzed in both shoots and roots and compared to the wild type. *GAD4* transcripts were found to be up-regulated (Figure 1B) and might be sufficient to explain the presence of GABA in the double mutant.

### An additional knock-out of *GABA-T* gene in the *gad1/2* double mutant caused higher GABA contents in shoots and roots

The GABA content of plant organs is not only determined by its synthesis. Its degradation by GABA-T activity also affects the accumulation of GABA, as was also discussed by Renault et al. (2010). We assumed that the low GABA concentration in *gad1/2* mutants would be elevated when breakdown of GABA is prevented due to the absence of GABA-T activity. Hence, we created a triple mutant by crossing the *gad1/2* double mutant to a *gaba-t* (*pop2-5*) mutant. *Pop2* mutants were previously shown to accumulate high GABA concentration in *A. thaliana* (Palanivelu et al., 2003; Ludewig et al., 2008; Renault et al., 2011). Homozygous knock-outs of all three genes of the triple mutant were verified by PCR (Figure S3). The *gad1/2 x pop2-5* triple mutant contained seven times more GABA than the wild-type and half as much compared with the *pop2-5* single mutant (just given for comparison) (Figure 2). It is likely that the triple mutant accumulates GABA during the course of growth for two reasons: firstly, the absence of GABA-T activity ensures that there is no GABA catabolism; secondly, the presence of decarboxylase activity by the remaining orthologs. In addition, higher GABA content in *pop2* single mutant compared to triple mutants is also not surprising since GAD1 and GAD2, the two prominent GADs, are active.

### Triple mutant plants are less susceptible to *Spodoptera littoralis* feeding

Due to the finding that higher GABA levels can affect insects (Ramputh and Bown, 1996; MacGregor et al., 2003; Bown et al., 2006) the influence of different endogenous GABA concentration *in planta* was investigated in parallel in an insect herbivore feeding assay. In contrast to former experiments (MacGregor et al., 2003; Bown et al., 2006), we did not look for feeding preferences but for insect performance on different mutant lines. Therefore, we carried out a bio-assay employing the three plant lines, wild type, g*ad1/2*, and g*ad1/2 x pop2-5* plants (Figure S4), and herbivorous larvae of the generalist lepidopteran species *Spodoptera littoralis*. We consciously did not include the GABA-enriched *pop2-5* line in these experiments. The reason for this was that higher constitutive GABA concentrations cause defects in regular plant growth. Among other effects, GABA levels, as found in *pop2* mutants, reduce gene expression levels of secreted proteins, in particular cell wall-related proteins, which in consequence affect cell elongation processes in reproductive and vegetative tissue (Palanivelu et al., 2003; Renault et al., 2010, 2011). Thus, we decided to work with plant lines with moderately increased GABA concentrations to avoid secondary effects on the whole plant-herbivore system.

**Figure 3:**
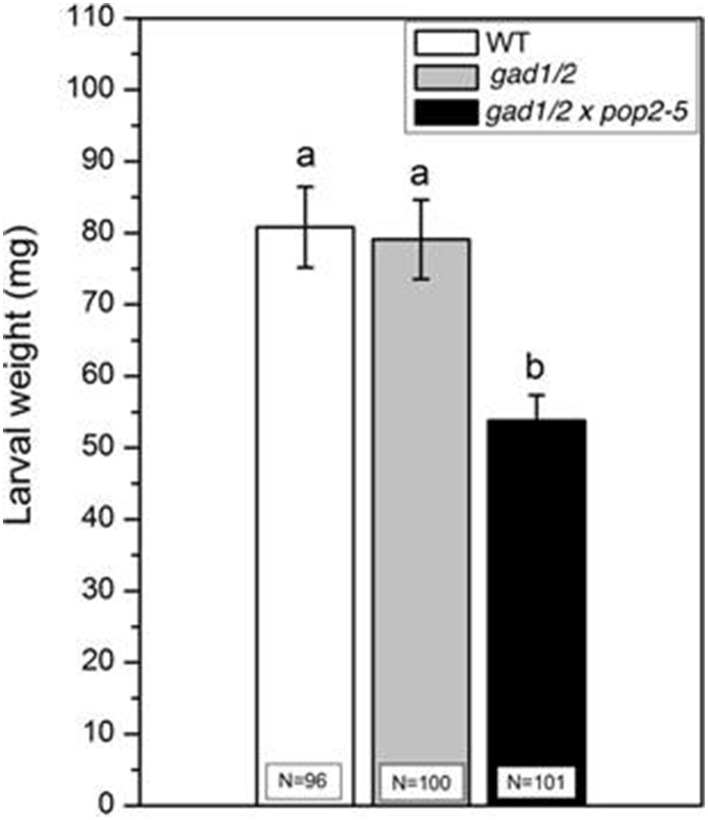
**Feeding assay of ***Spodoptera littoralis*** larvae on ***Arabidopsis*** wild-type (WT), ***gad1/2*** and ***gad1/2*** x ***pop2-5*** plants**. *S. littoralis* 1st instar larvae were pre-weighed and three larvae were placed on each plant. The larval weight (mean ± SE) was measured after 7 days of feeding. The total number of larvae weighed (N) is indicated in the bars. Experiments were repeated four times independently. Statistically significant differences between WT and GABA mutant plants after feeding were analyzed by One-way ANOVA (*p* < 0.05, SNK-test) and are indicated by different letters.

**Figure 4:**
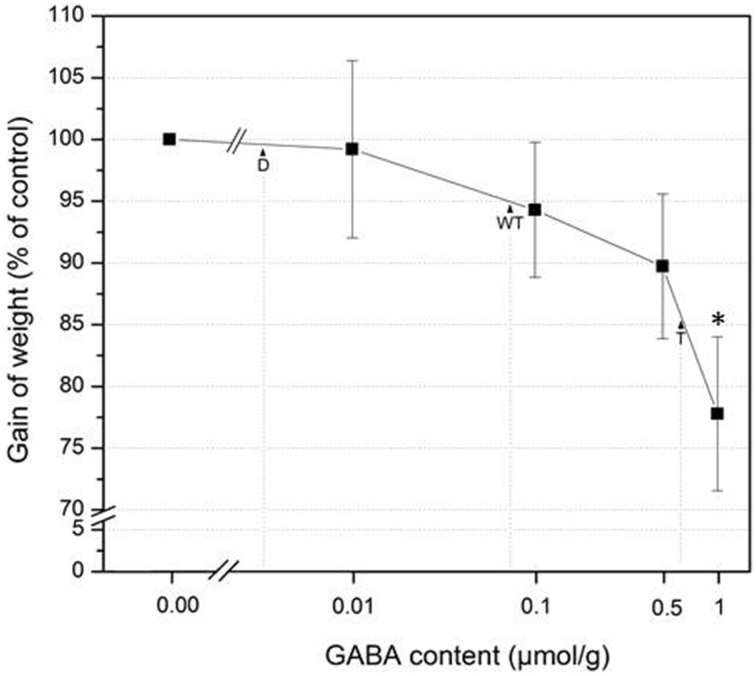
**Effects of GABA on ***S. littoralis g***rowth**. Mean (± SE, *n* = 18–20) gain of weight of 2nd instar *S. littoralis* larvae feeding on artificial diet containing 0, 0.01, 0.1, 0.5, and 1 μmol GABA /g^−1^. Larval weight was determined after 7 days of feeding and the increase in weight correlated with the starting weight. Gain of weight was calculated relative to the weight gained after control treatment without GABA (=100%). Statistically significant differences between the control and the respective treatment was analyzed by *t*-test (for each concentration separately), ^*^P = < 0.05. D double mutant (*gad1/2*), WT wild type, T triple mutant (*gad1/2 x pop2-5*).

While *S. littoralis* larvae feeding on *gad1/2* mutant plants showed the same increase in body weight as on wild type, the larvae feeding on g*ad1/2 x pop2-5* plants gained significantly less weight (Figure 3). The constitutive accumulation of GABA over time in this mutant (Figure 2) might contribute to the enhanced resistance against *S. littoralis* feeding. Interestingly, lower GABA level in the *gad1/2* mutant did not result in an altered feeding behavior of *S. littoralis* larvae compared to the wild type (Figure 3) suggesting that this insect species can tolerate some basic level of the defensive compound GABA. To follow up this idea, 2nd instar *S. littoralis* larvae were reared on an artificial diet containing different amounts of GABA (Figure 4). Concentrations were chosen between 0 and 1 μmol GABA (g diet)^−1^; these concentrations covered the GABA levels determined for the investigated wild type and mutant lines (Figure 2). Interestingly, lower concentrations of GABA between 0 and 0.08 μmol g^−1^, which resembled the constitutive GABA concentration in *A. thaliana* Col-0 wild type plants, did not significantly affect *S. littoralis* larvae growth (Figure 4). The increase in larval weight is reduced about 5 % compared to water treatment. This observation suggests that *S. littoralis* does indeed have a certain tolerance to GABA in its food source. A significant decrease in growth was observed for a GABA content of 1 μmol g^−1^; here the larvae gained 23% less weight compared to the control (Figure 4). Thus, these results can explain the *S. littoralis* feeding behavior on the different GABA mutant lines where the GABA concentration of both wild type and the *gad1/2* mutant did not cause any growth inhibition (Figure 3) but, in contrast, the GABA concentration of the g*ad1/2 x pop2-5* triple mutant induced a decrease in growth of about 15%. However, this alone cannot explain the results shown in Figure 3, but an increased GABA level very likely contributes to the whole array of defenses against *S. littoralis*.

A similar finding for a species-specific tolerance has been described for *S. littoralis* that fed on *Nicotiana attenuata* mutant plants (irMPK4 × irCOI1), where a jasmonate-independent defense pathway could not inhibit growth of *S. littoralis* larvae in contrast to larvae of *Manduca sexta* (Hettenhausen et al., 2013).

### *Spodoptera littoralis* feeding- and wounding-induced jasmonate induction is not affected in GABA mutants

Knowing that many plant defense reactions against herbivorous insects are regulated by jasmonates (Wasternack, 2007; Mithöfer et al., 2009) we decided to further investigate the contribution and involvement of this phytohormone class on GABA accumulation. Thus, the levels of jasmonic acid (JA) and its bioactive derivative, (+)-7-*iso*-jasmonoyl-*L*-isoleucine (JA-Ile) (Fonseca et al., 2009), were determined in *Arabidopsis* wild type and the GABA mutant plants upon herbivore treatment (Figures S5A,B).

**Figure 5:**
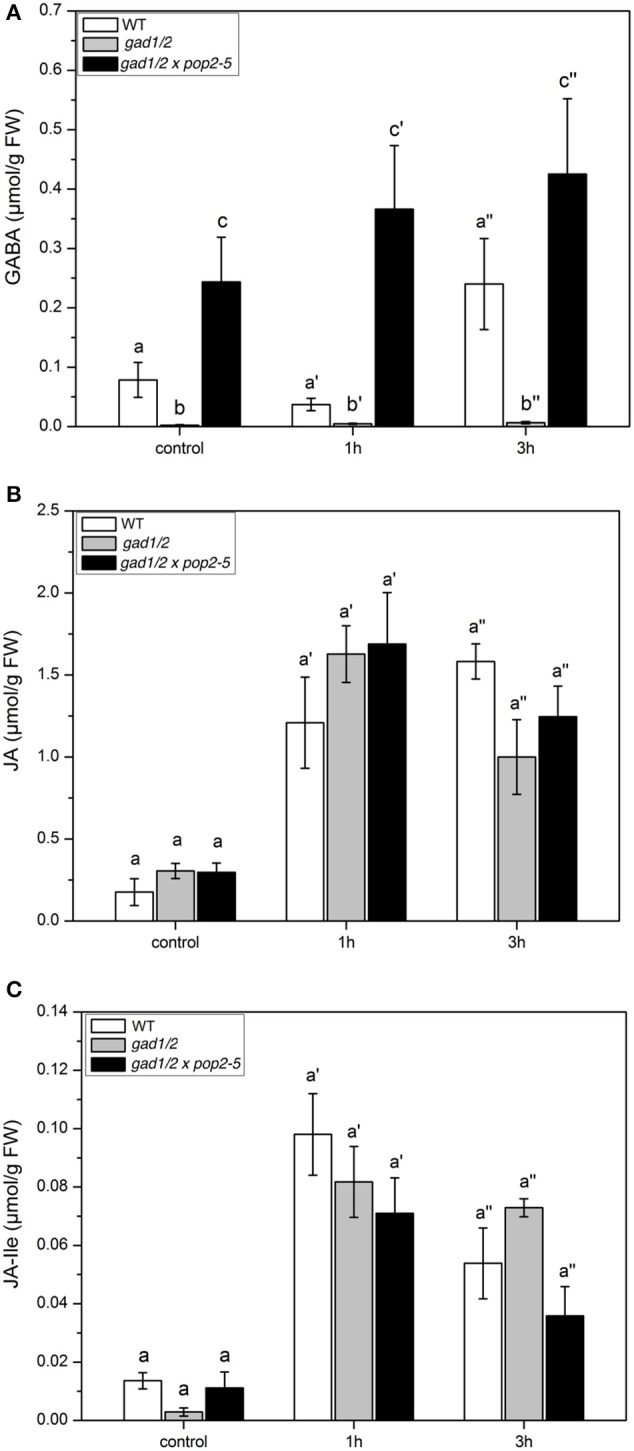
**GABA and Jasmonate levels upon ***Spodoptera littoralis*** herbivory in ***Arabidopsis*** wild-type (WT), ***gad1/2*** and ***gad1/2*** x ***pop2-5*** plants**. Mean (± SE, *n* = 10) levels of GABA **(A)**, JA **(B)**, and JA-Ile **(C)** in Col-0 WT, *gad1/2* (gray) and *gad1/2* x *pop2-5* (black) plants after *S. littoralis* feeding (2nd instar) for 1 and 3 h. Hormone and GABA levels were measured only from local *S. littoralis* fed leaves. Untreated leaves from untreated plants were used as controls. Statistically significant differences between hormones in WT and GABA mutant plants after feeding were analyzed by One-way ANOVA (*p* < 0.05, SNK-test) and are indicated by different letters.

As shown in Figure 5A, the differences in basic GABA content present in the three plant lines are obvious. In wild type and in the triple mutant GABA level increased over time. The content of JA and JA-Ile also clearly increased due to larvae feeding but no significant differences were detectable between wild-type and the two mutant lines (Figures 5B,C). Thus, the different levels of GABA did not affect the jasmonate level.

**Figure 6:**
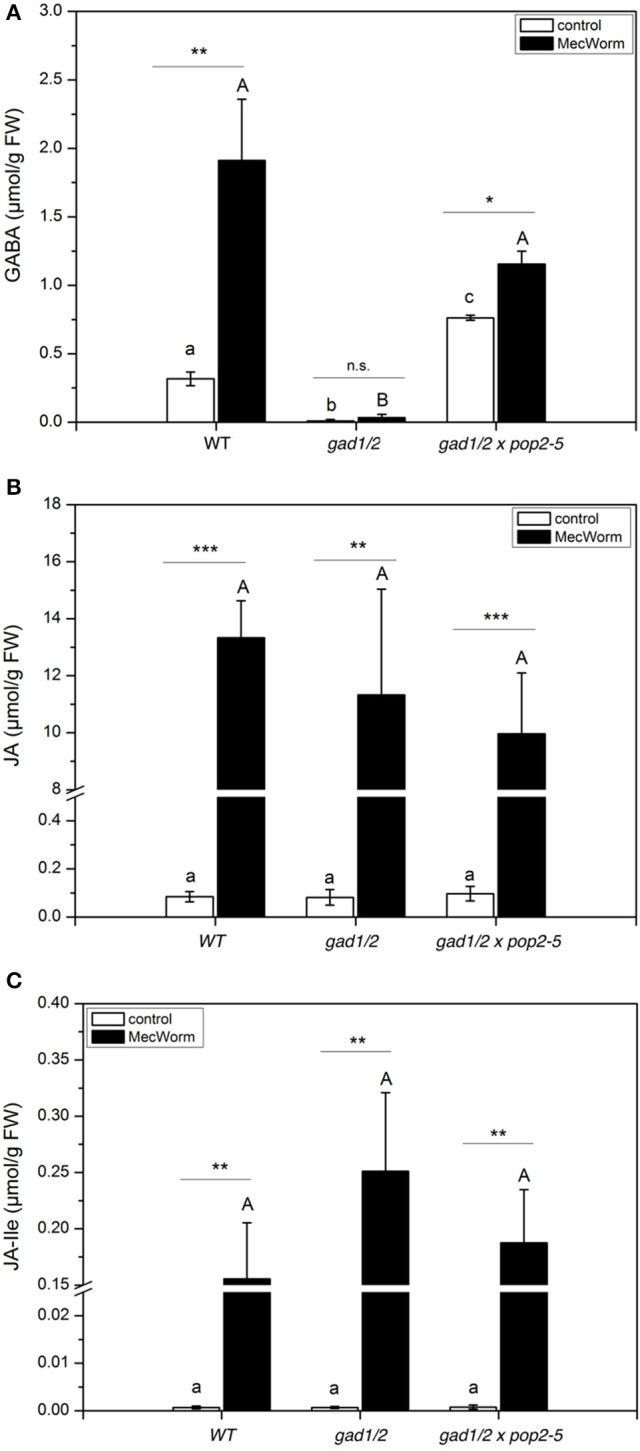
**GABA and Jasmonate levels upon MecWorm treatment in ***Arabidopsis*** wild-type (WT), ***gad1/2*** and ***gad1/2*** x ***pop2-5*** plants**. Mean (± SE, *n* = 6) levels of GABA **(A)**, JA **(B)**, and JA-Ile **(C)** were determined in control plants and 3 h after treatment (black). Hormone and GABA levels were measured only from treated leaves. Untreated leaves from untreated plants were used as controls. Statistically significant differences between hormones in different mutants were analyzed by One-way ANOVA (*p* < 0.05, SNK-test) and are indicated by different letters. Statistical significant differences between control and treated plants were analyzed by *t*-test, ^*^P ≤ 0.05, ^**^P ≤ 0.01, ^***^P ≤ 0.001.

Insect herbivory is a combination of two events, firstly the wounding of plant tissues and secondly the introduction of insect-derived compounds that come in contact with the tissues during the feeding process (Mithöfer and Boland, 2008). Using a robotic caterpillar, MecWorm (Figures S5C–H), we are able to mimic the behavior of a feeding *S. littoralis* larva in order to investigate the impact of the isolated wounding process without the contribution of insect-derived compounds (Mithöfer et al., 2005). As shown in Figure 6, MecWorm treatment alone caused the accumulation of GABA in wild-type plants. Wounding disrupts cell structure and releases the acidic vacuole content. As shown for carrot suspension cells, acidic pH values stimulate GAD activity *in vivo*, and as a consequence thereof, the generation and accumulation of GABA (Carroll et al., 1994). Compared with insect feeding (Figure 5A), MecWorm wounding caused an eight-fold higher GABA accumulation in wild-type plants due to the facts that more leaf material was wounded and, in addition, the wounded leaf material was not completely removed as by ingestion during insects feeding. As expected, in the knock out plant *gad1/2* no GABA accumulated (Figure 6A). Jasmonate levels increased significantly upon wounding; however, the amount of JA and JA-Ile in the controls and in the treated plants was similar, independent on the plant lines (Figures 6B,C).

### Wounding induces GABA accumulation in adjacent leaves

**Figure 7:**
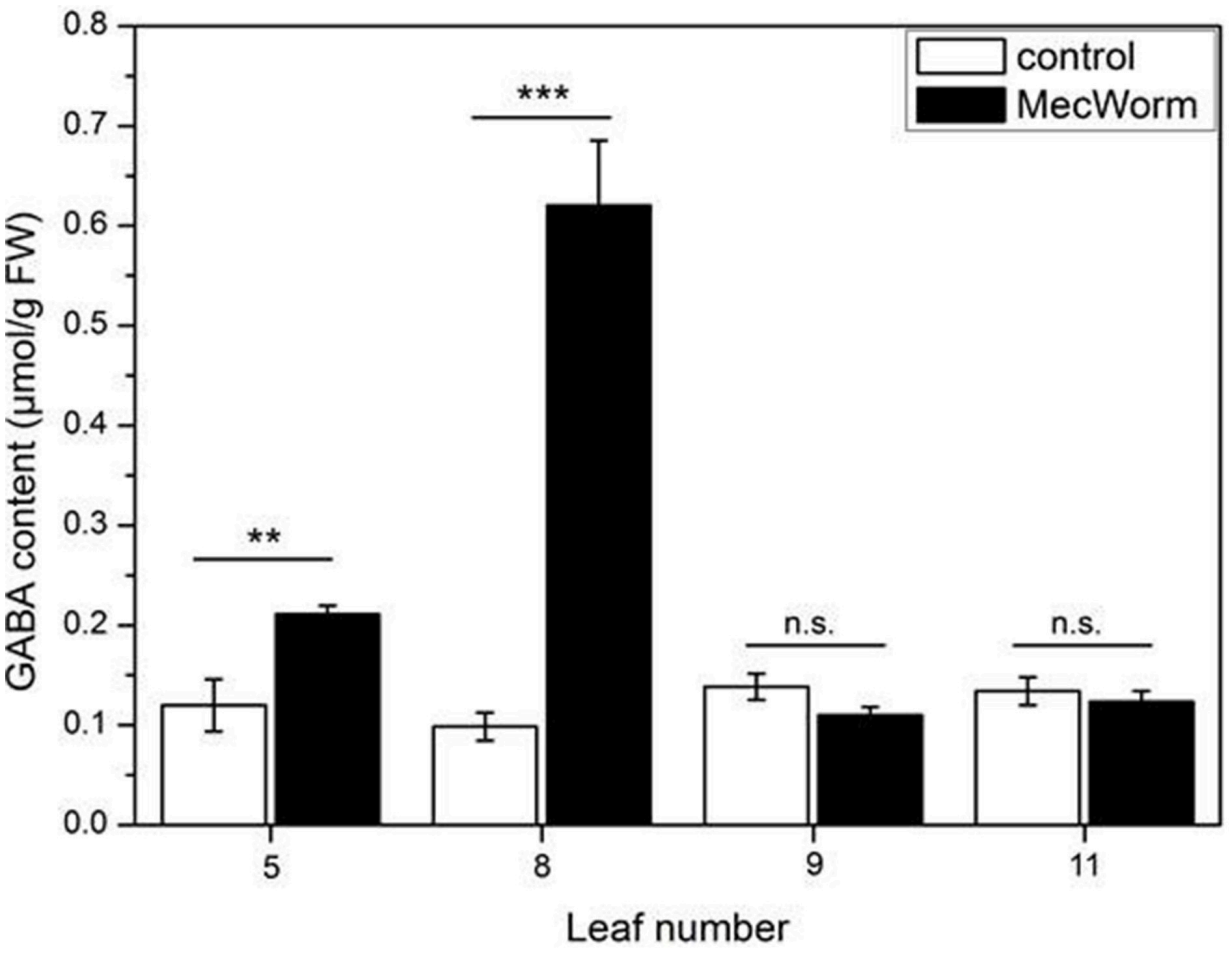
**Accumulation of GABA in individual ***Arabidopsis*** leaves after MecWorm treatment**. Mean (± SE, *n* = 5) levels of GABA were determined in individual leaves of untreated control plants and plants after treatment for 1 h with MecWorm. In treated plants, leaf 8 was subjected to mechanical damage and systemic leaves 5, 9, and 11, and treated leaf 8 were analyzed for GABA level. Statistically significant differences between the GABA level in the same leaf of the control and treated plant were analyzed by *t*-test (for each leaf separately, *p* < 0.05, Mann-Whitney-U test), ^**^P ≤ 0.01; ^***^P ≤ 0.001.

An interesting feature of the GABA-forming GAD enzyme is its activation in acidic conditions, whereas under neutral conditions the activity depends on Ca^2+^/calmodulin (Snedden et al., 1995; Bown et al., 2006). Thus, wounding and the accompanying acidification of the cytosol can explain GABA accumulation in the treated, local leaf. In *Asparagus sprengeri* (Regel) mesophyll cells a Ca^2+^-dependent activation of GAD could be demonstrated (Cholewa et al., 1997). Knowing that wounding and herbivory can also stimulate a systemic increase of the cytosolic Ca^2+^ concentration (Kiep et al., 2015), the systemic accumulation of GABA was investigated upon wounding of a defined leaf with MecWorm. As shown in Figure 7, mechanical damage of leaf 8 did not only cause a significant increase of GABA concentration in the local leaf but also in the adjacent leaf 5, which is directly connected to leaf 8 (Farmer et al., 2013). Although no response was detected in other leaves, this result strongly suggests that the induced Ca^2+^ increase in non-wounded tissue can trigger the activity of GAD, supporting *in vivo* the statement of Snedden et al. (1995) that systemic GABA synthesis might depend on Ca^2+^ signaling.

### GABA elevation is jasmonate independent

**Figure 8:**
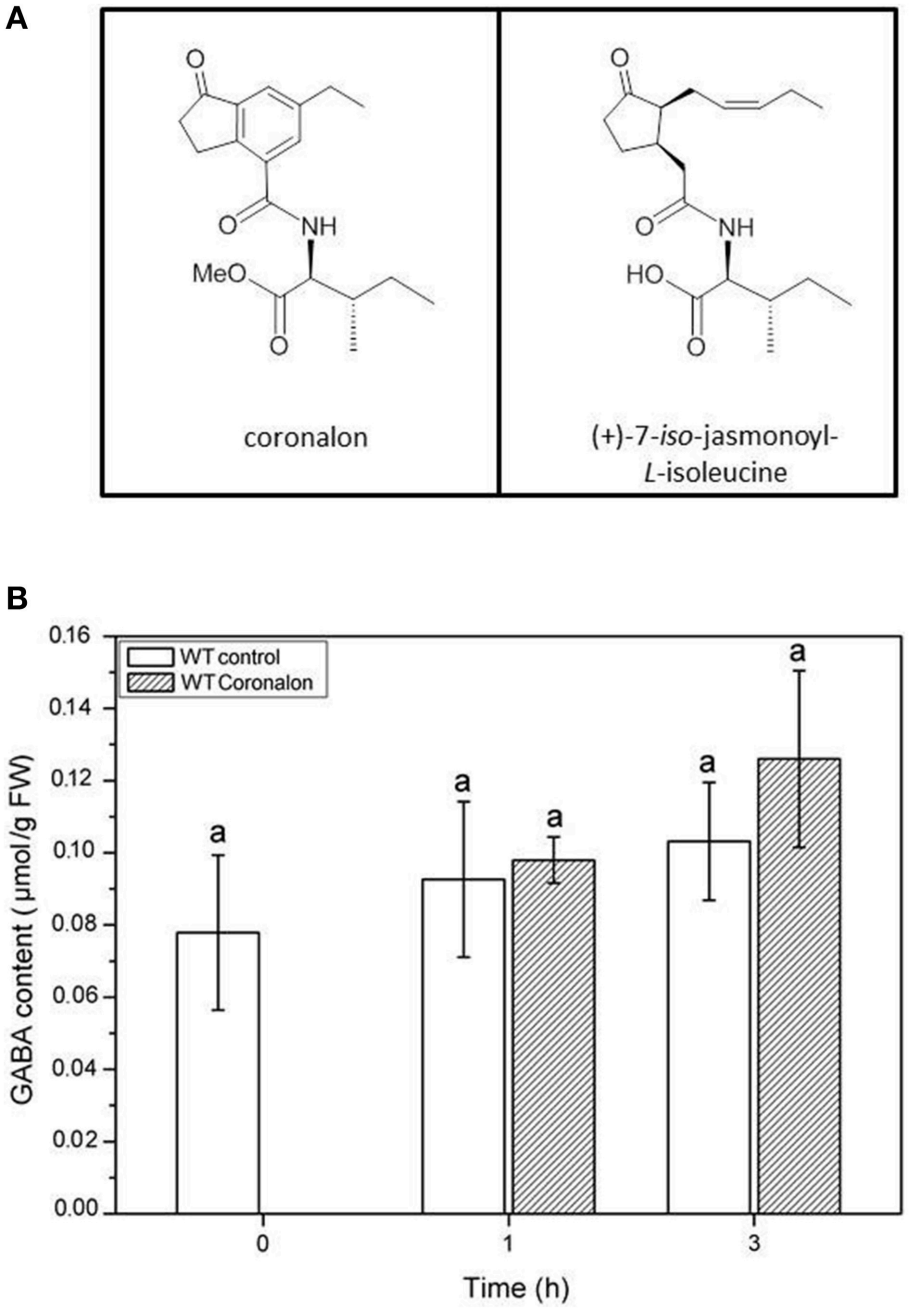
**Accumulation of GABA after coronalon treatment in wild-type (WT) plants. (A)** Structures of (+)-7-*iso*-jasmonoyl-L-isoleucine (JA-Ile) and structural mimic 6-ethyl indanoyl isoleucine (coronalon). **(B)** Mean (± SE, *n* = 10) levels of GABA were determined after spray with solvent control (0.1% ethanol, white) or 50 μM coronalon 1 and 2 h after treatment. Statistically significant differences between the treatments were analyzed by One-way ANOVA (*p* < 0.05, SNK-test) and are indicated by different letters.

**Figure 9:**
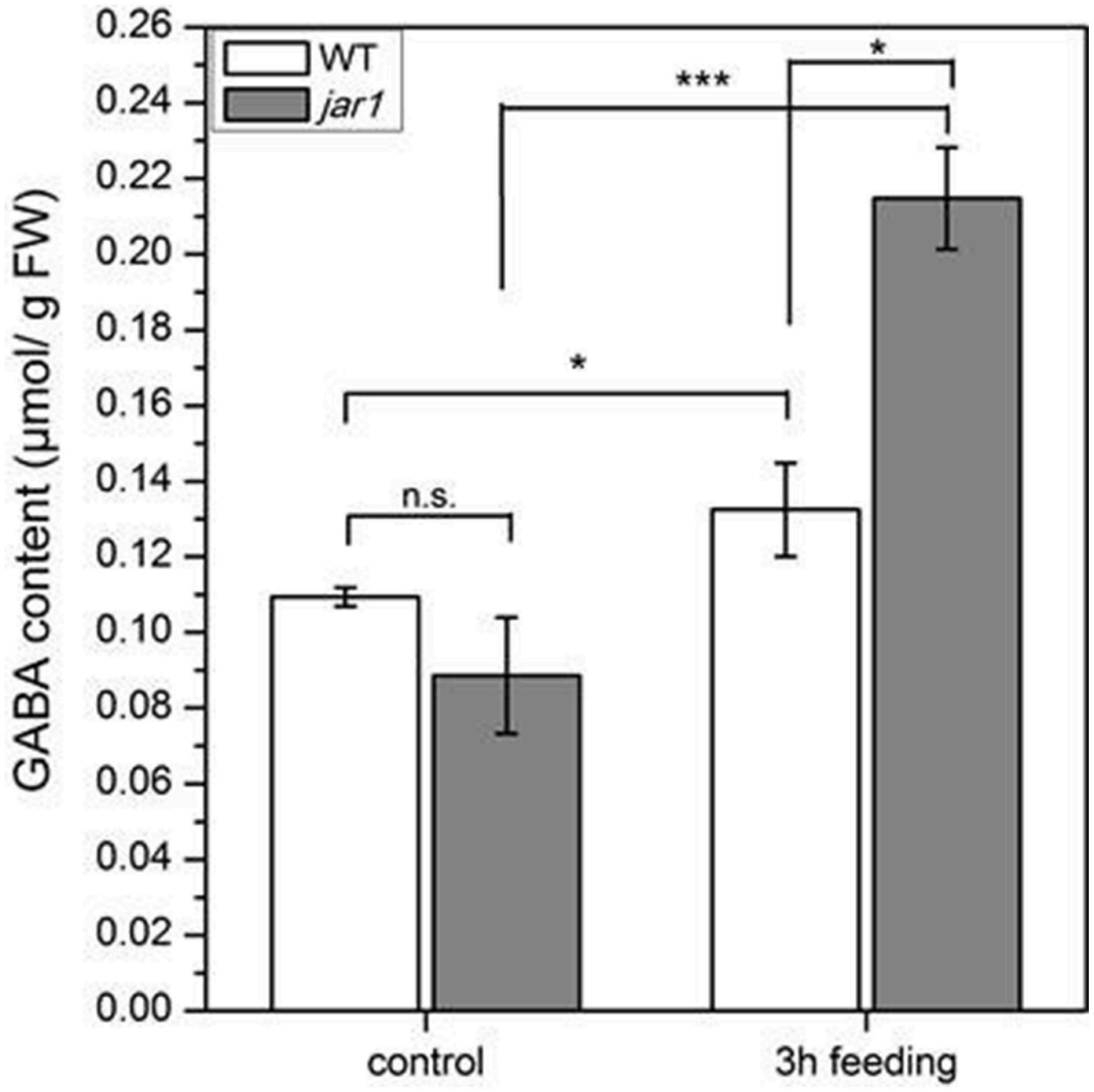
**Accumulation of GABA after ***S. littoralis*** feeding in wild-type (WT) and ***jar1*** plants**. Mean (± SE, *n* = 6) levels of GABA were determined after a feeding period of 3 h. Hormone and GABA levels were measured only from local *S. littoralis*-fed leaves. Untreated leaves from untreated plants were used as controls. Statistically significant differences between the treatments were analyzed by *t*-test, ^*^P ≤ 0.05, ^***^P ≤ 0.001.

To further investigate whether the accumulation of GABA might be induced by jasmonates, we treated *Arabidopsis* wild-type plants with the synthetic JA-Ile mimic coronalon (Figure 8A) that has been shown to induce all typical JA-Ile effects (Schüler et al., 2004; Svoboda and Boland, 2010; Nakamura et al., 2014). As can be seen in Figure 8B, no changes in GABA concentration were detectable within 3 h after treatment with 50 μM coronalon, while JA-biosynthesis and JA-responsive genes were induced indicating a sufficient concentration of coronalon (Figure S6). This result is clearly in contrast to the results obtained in wild-type plants where GABA accumulation was detected upon herbivory (Figure 5A) or mechanical wounding (Figure 6A) within the same period, indicating that GABA accumulation is not jasmonate dependent. In order to support this result, we performed an additional experiment where wild-type *Arabidopsis* and a jasmonate signaling mutant, *jar1* that is unable to generate JA-Ile (Staswick et al., 2002), were treated with *S. littoralis* larvae. Whereas in wild-type and *jar1* control plants the level of GABA was the same, after 3 h of feeding in wild-type as well as in *jar1* plants a significant higher content of GABA was detected compared to the respective controls (Figure 9). This was an expected result because the feeding process causes GABA accumulation (Figure 5A). Strikingly, in this particular experiment the induced GABA content in the WT was lower compared to former experiments. This can be explained by different feeding behavior of the larvae which is usually observed for this kind of bio assays demonstrating the need of simultaneously performed controls. More interesting is the finding that in *jar1* plants a significant increase of GABA could be measured compared with wild type plants (Figure 9). On the one hand this shows again that jasmonate-based signaling is not involved in GABA accumulation and on the other hand that on defense-impaired *jar1* plants more GABA could be generated very likely because the larvae fed more.

## Conclusion

The non-proteinogenic amino acid γ-aminobutyric acid, GABA, is widespread in eukaryotes including invertebrates, where it activates Cl^−^ -channels at neuromuscular junctions. For plants, various physiological role(s) for GABA are still under discussion. Here, experimental evidence based on GABA-reduced and GABA-enriched *Arabidopsis thaliana* mutants demonstrates that wounding of plant tissue and cell disruption caused by feeding insects is sufficient to induce rapid, jasmonate-independent GABA synthesis and accumulation. When ingested the elevated GABA levels become toxic for the insect. Similar to the tissue- and cell disruption-mediated formation of toxic isothiocyanates from glucosinolates and hydrogen cyanide (HCN) from cyanogenic glucosides (Mithöfer and Boland, 2012), respectively, GABA formation from glutamate is a component in a plant's first-line of general, rapid defense against invertebrate pests.

## Author contributions

SS: Developed research concept; carried out all herbivory-related experiments, wrote the paper. MR: Analyzed the phytohormones and GABA. DM: Developed research concept; created and analyzed the mutants, wrote the paper. FL: Developed research concept; wrote the paper. AM: Developed research concept; wrote the paper.

### Conflict of interest statement

The authors declare that the research was conducted in the absence of any commercial or financial relationships that could be construed as a potential conflict of interest.

## References

[B1] Alonso J. M. Stepanova A. N. Leisse T. J. Kim C. J. Chen H. M. Shinn P.. (2003). Genome-wide insertional mutagenesis of *Arabidopsis thaliana*. Science 301, 653–657. 10.1126/science.1086391 12893945

[B2] Bergomaz R. Boppre M. (1986). A simple instant diet for rearing arctiidae and other moths. J. Lepid. Soc. 40, 131–137.

[B3] Bouché N. Fait A. Bouchez D. Møller S. G. Fromm H. (2003). Mitochondrial succinic-semialdehyde dehydrogenase of the gamma-aminobutyrate shunt is required to restrict levels of reactive oxygen intermediates in plants. Proc. Natl. Acad. Sci. U.S.A. 100, 6843–6848. 10.1073/pnas.1037532100 12740438 PMC164534

[B4] Bouché N. Fromm H. (2004). GABA in plants: just a metabolite? Trends Plant Sci. 9, 110–115. 10.1016/j.tplants.2004.01.006 15003233

[B5] Bouchereau A. Aziz A. Larher F. Martin-Tanguy J. (1999). Polyamines and environmental challenges: recent development. Plant Sci. 140, 103–125. 10.1016/S0168-9452(98)00218-0

[B6] Bown A. W. Hall D. E. MacGregor K. B. (2002). Insect footsteps on leaves stimulate the accumulation of 4-aminobutyrate and can be visualized through increased chlorophyll fluorescence and superoxide production. Plant Physiol. 129, 1430–1434. 10.1104/pp.006114 12177456 PMC1540246

[B7] Bown A. W. Macgregor K. B. Shelp B. J. (2006). Gamma-aminobutyrate: defense against invertebrate pests? Trends Plant Sci. 11, 424–427. 10.1016/j.tplants.2006.07.002 16890474

[B8] Breitkreuz K. E. Allan W. L. Van Cauwenberghe O. R. Jakobs C. Talibi D. Andre B.. (2003). A novel gamma-hydroxybutyrate dehydrogenase: identification and expression of an *Arabidopsis* cDNA and potential role under oxygen deficiency. J. Biol. Chem. 278, 41552–41556. 10.1074/jbc.M305717200 12882961

[B9] Breitkreuz K. E. Shelp B. J. (1995). Subcellular compartmentation of the 4-aminobutyrate shunt in protoplasts from developing soybean cotyledons. Plant Physiol. 108, 99–103. 12228455 10.1104/pp.108.1.99 PMC157309

[B10] Carroll A. D. Fox G. G. Laurie S. Phillips R. Ratcliffe R. G. Stewart G. R. (1994). Ammonium assimilation and the role of γ-aminobutyric acid in pH homeostasis in carrot cell suspensions. Plant Physiol. 106, 513–520. 12232346 10.1104/pp.106.2.513 PMC159556

[B11] Cholewa E. Bown A. W. Cholewinski A. J. Shelp B. J. Snedden W. A. (1997). Cold-shock-stimulated γ-aminobutyric acid synthesis is mediated by an increase in cytosolic Ca2+, not by an increase in cytosolic H+. Can. J. Bot. 75, 375–382. 10.1139/b97-040

[B12] Clark S. M. Di Leo R. Van Cauwenberghe O. R. Mullen R. T. Shelp B. J. (2009). Subcellular localization and expression of multiple tomato gamma-aminobutyrate transaminases that utilize both pyruvate and glyoxylate. *J. Exp*. Bot. 60, 3255–3267. 10.1093/jxb/erp161 PMC2718222 19470656

[B13] Fait A. Fromm H. Walter D. Galili G. Fernie A. R. (2008). Highway or byway: the metabolic role of the GABA shunt in plants. Trends Plant Sci. 13, 14–19. 10.1016/j.tplants.2007.10.005 18155636

[B14] Fait A. Yellin A. Fromm H. (2005). GABA shunt deficiencies and accumulation of reactive oxygen intermediates: insight from *Arabidopsis* mutants. FEBS Lett. 579, 415–420. 10.1016/j.febslet.2004.12.004 15642352

[B15] Farmer E. Mousavi S. Lenglet A. (2013). Leaf numbering for experiments on long distance signalling in *Arabidopsis*. Protoc. Exch. 10.1038/protex.2013.071

[B16] Fonseca S. Chini A. Hamberg M. Adie B. Porzel A. Kramell R. (2009). (+)-7-*iso*-Jasmonoyl-L-isoleucine is the endogenous bioactive jasmonate. *Nat. Chem. Biol* 5, 344–350. 10.1038/nchembio.161 19349968

[B17] Hettenhausen C. Baldwin I. T. Wu J. (2013). *Nicotiana attenuata* MPK4 suppresses a novel jasmonic acid (JA) signaling-independent defense pathway against the specialist insect *Manduca sexta*, but is not required for the resistance to the generalist *Spodoptera littoralis*. New Phytol. 199, 787–799. 10.1111/nph.12312 23672856 PMC4996321

[B18] Hruz T. Laule O. Szabo G. Wessendorp F. Bleuler S. Oertle L.. (2008). Genevestigator V3: a reference expression database for the meta-analysis of transcriptomes. Adv. Bioinformatics 2008:420747. 10.1155/2008/420747 19956698 PMC2777001

[B19] Huang T. Jander G. de Vos M. (2011). Non-protein amino acids in plant defense against insect herbivores: representative cases and opportunities for further functional analysis. Phytochemistry 72, 1531–1537. 10.1016/j.phytochem.2011.03.019 21529857

[B20] Kiep V. Vadassery J. Lattke J. Maaß J.-P. Boland W. Peiter E.. (2015). Systemic cytosolic Ca^2+^ elevation is activated upon wounding and herbivory in *Arabidopsis*. New Phytol. 207, 996–1004. 10.1111/nph.13493 25996806

[B21] Kinnersley A. M. Turano F. J. (2000). Gamma aminobutyric acid (GABA) and plant responses to stress. *Crit. Rev*. Plant Sci. 19, 479–509. 10.1016/S0735-2689(01)80006-X

[B22] Kleinboelting N. Huep G. Kloetgen A. Viehoever P. Weisshaar B. (2012). GABI-Kat SimpleSearch: new features of the *Arabidopsis thaliana* T-DNA mutant database. Nucleic Acids Res. 40, D1211–D1215. 10.1093/nar/gkr1047 22080561 PMC3245140

[B23] Kramell R. Schmidt J. Schneider G. Sembdner G. Schreiber K. (1988). Synthesis of N-(Jasmonyl)amino acid conjugates. Tetrahedron 44, 5791–5807. 10.1016/S0040-4020(01)81437-X

[B24] Logemann J. Schell J. Willmitzer L. (1987). Improved method for the isolation of RNA from plant tissues. *Anal*. Biochem. 163, 16–20. 10.1016/0003-2697(87)90086-8 2441623

[B25] Ludewig F. Hüser A. Fromm H. Beauclair L. Bouché N. (2008). Mutants of GABA-Transaminase (*POP2*) suppress the severe phenotype of succinic semialdehyde dehydrogenase (*ssadh*) mutants in *Arabidopsis*. PLoS ONE 3:e3383. 10.1371/journal.pone.0003383 18846220 PMC2557145

[B26] MacGregor K. B. Shelp B. J. Peiris S. Bown A. W. (2003). Overexpression of glutamate decarboxylase in transgenic tobacco plants deters feeding by phytophagous insect larvae. J. Chem. Ecol. 29, 2177–2182. 10.1023/A:1025650914947 14584684

[B27] Marchler-Bauer A. Lu S. Anderson J. B. Chitsaz F. Derbyshire M. K. Deweese-Scott C.. (2011). CDD: a conserved domain database for the functional annotation of proteins. Nucleic Acids Res. 39, D225–D229. 10.1093/nar/gkq1189 21109532 PMC3013737

[B28] McLean M. Yevtushenko D. Deschene A. Van Cauwenberghe O. Makhmoudova A. Potter J. (2003). Overexpression of glutamate decarboxylase in transgenic tobacco plants confers resistance to the northern root-knot nematode. *Mol*. Breed. 11, 277–285. 10.1023/A:1023483106582

[B29] Michaeli S. Fait A. Lagor K. Nunes-Nesi A. Grillich N. Yellin A.. (2011). A mitochondrial GABA permease connects the GABA shunt and the TCA cycle, and is essential for normal carbon metabolism. Plant J. 67, 485–498. 10.1111/j.1365-313X.2011.04612.x 21501262

[B30] Mirabella R. Rauwerda H. Struys E. A. Jakobs C. Triantaphylidés C. Haring M. A.. (2008). The *Arabidopsis her1* mutant implicates GABA in E-2-hexenal responsiveness. Plant J. 53, 197–213. 10.1111/j.1365-313X.2007.03323.x 17971036

[B31] Mithöfer A. Boland W. (2008). Recognition of herbivory-associated molecular patterns. Plant Physiol. 146, 825–831. 10.1104/pp.107.113118 18316636 PMC2259064

[B32] Mithöfer A. Boland W. (2012). Plant defense against herbivores: chemical aspects. *Annu. Rev*. Plant Biol. 63, 431–450. 10.1146/annurev-arplant-042110-103854 22404468

[B33] Mithöfer A. Boland W. Maffei M. E. (2009). Chemical ecology of plant–insect interactions, in Annual Plant Reviews: Molecular Aspects of Plant Disease Resistance, ed Parker J. (Chichester: Wiley-Blackwell), 261–291.

[B34] Mithöfer A. Wanner G. Boland W. (2005). Effects of feeding *Spodoptera littoralis* on lima bean leaves. II. Continuous mechanical wounding resembling insect feeding is sufficient to elicit herbivory-related volatile emission. Plant Physiol. 137, 1160–1168. 10.1104/pp.104.054460 15728342 PMC1065415

[B35] Molina-Rueda J. J. Pascual M. B. Pissarra J. Gallardo F. (2015). A putative role for gamma-aminobutyric acid (GABA) in vascular development in pine seedlings. Planta 241, 257–267. 10.1007/s00425-014-2157-4 25183257

[B36] Nakamura Y. Paetz C. Brandt W. David A. Rendon-Anaya M. Herrera-Estrella A. (2014). Synthesis of 6-substituted 1-oxoindanoyl isoleucine conjugates and modeling studies with the COI1-JAZ co-receptor complex of lima bean. *J. Chem*. Ecol. 40, 687–699. 10.1007/s10886-014-0469-2 25008776

[B37] Palanivelu R. Brass L. Edlund A. F. Preuss D. (2003). Pollen tube growth and guidance is regulated by *POP2*, an *Arabidopsis* gene that controls GABA levels. Cell 114, 47–59. 10.1016/S0092-8674(03)00479-3 12859897

[B38] Ramesh S. A. Tyerman S. D. Xu B. Bose J. Kaur S. Conn V.. (2015). GABA signalling modulates plant growth by directly regulating the activity of plant-specific anion transporters. Nat. Commun. 6, 7879. 10.1038/ncomms8879 26219411 PMC4532832

[B39] Ramputh A. I. Bown A. W. (1996). Rapid [gamma]-aminobutyric acid synthesis and the inhibition of the growth and development of oblique-banded leaf-roller larvae. Plant Physiol. 111, 1349–1352. 12226367 10.1104/pp.111.4.1349 PMC161023

[B40] Renault H. El Amrani A. Palanivelu R. Updegraff E. P. Yu A. Renou J. P.. (2011). GABA accumulation causes cell elongation defects and a decrease in expression of genes encoding secreted and cell wall-related proteins in *Arabidopsis thaliana*. Plant Cell Physiol. 52, 894–908. 10.1093/pcp/pcr041 21471118 PMC3093128

[B41] Renault H. Roussel V. El Amrani A. Arzel M. Renault D. Bouchereau A.. (2010). The *Arabidopsis pop2-1* mutant reveals the involvement of GABA transaminase in salt stress tolerance. BMC Plant Biol. 10:20. 10.1186/1471-2229-10-20 20122158 PMC2825238

[B42] Scholz S. S. Vadassery J. Heyer M. Reichelt M. Bender K. W. Snedden W. A.. (2014). Mutation of the *Arabidopsis* calmodulin-like protein CML37 deregulates the jasmonate pathway and enhances susceptibility to herbivory. Mol. Plant 7, 1712–1726. 10.1093/mp/ssu102 25267731

[B43] Schüler G. Mithöfer A. Baldwin I. T. Berger S. Ebel J. Santos J. G.. (2004). Coronalon: a powerful tool in plant stress physiology. FEBS Lett. 563, 17–22. 10.1016/S0014-5793(04)00239-X 15063716

[B44] Shelp B. J. Allan W. L. Faure D. (2009). Role of γ -aminobutyrate and γ-hydroxybutyrate in plant communication, in Plant-Environment Interactions, ed Baluška F. (Berlin; Heidelberg: Springer-Verlag), 73–84. 10.1007/978-3-540-89230-4_4

[B45] Shelp B. J. Bown A. W. Faure D. (2006). Extracellular gamma-aminobutyrate mediates communication between plants and other organisms. Plant Physiol. 142, 1350–1352. 10.1104/pp.106.088955 17151138 PMC1676054

[B46] Shelp B. J. Bown A. W. McLean M. D. (1999). Metabolism and functions of gamma-aminobutyric acid. Trends Plant Sci. 4, 446–452. 10.1016/S1360-1385(99)01486-7 10529826

[B47] Shelp B. J. Bozzo G. G. Trobacher C. P. Zarei A. Deyman K. L. Brikis C. J. (2012a). Hypothesis/review: contribution of putrescine to 4-aminobutyrate (GABA) production in response to abiotic stress. Plant Sci. 193–194, 130–135. 10.1016/j.plantsci.2012.06.001 22794926

[B48] Shelp B. J. Bozzo G. G. Zarei A. Simpson J. P. Trobacher C. P. Allan W. L. (2012b). Strategies and tools for studying the metabolism and function of γ-aminobutyrate in plants. II. Integrated analysis. Botany 90, 781–793. 10.1139/b2012-041

[B49] Snedden W. A. Arazi T. Fromm H. Shelp B. J. (1995). Calcium/calmodulin activation of soybean glutamate decarboxylase. Plant Physiol. 108, 543–549. 12228492 10.1104/pp.108.2.543 PMC157373

[B50] Staswick P. E. Tiryaki I. Rowe M. L. (2002). Jasmonate response locus *JAR1* and several related *Arabidopsis* genes encode enzymes of the firefly luciferase superfamily that show activity on jasmonic, salicylic, and indole-3-acetic acids in an assay for adenylation. Plant Cell 14, 1405–1415. 10.1105/tpc.000885 12084835 PMC150788

[B51] Svoboda J. Boland W. (2010). Plant defense elicitors: analogues of jasmonoyl-isoleucine conjugate. Phytochemistry 71, 1445–1449. 10.1016/j.phytochem.2010.04.027 20570297

[B52] Turano F. J. Fang T. K. (1998). Characterization of two glutamate decarboxylase cDNA clones from *Arabidopsis*. Plant Physiol. 117, 1411–1421. 10.1104/pp.117.4.1411 9701597 PMC34905

[B53] Vadassery J. Reichelt M. Hause B. Gershenzon J. Boland W. Mithöfer A. (2012). CML42-mediated calcium signaling coordinates responses to Spodoptera herbivory and abiotic stresses in *Arabidopsis*. Plant Physiol. 159, 1159–1175. 10.1104/pp.112.198150 22570470 PMC3387702

[B54] Wallace W. Secor J. Schrader L. E. (1984). Rapid accumulation of γ-aminobutyric acid and alanine in soybean leaves in response to an abrupt transfer to lower temperature, darkness, or mechanical manipulation. Plant Physiol. 75, 170–175. 10.1104/pp.75.1.170 16663565 PMC1066856

[B55] Wasternack C. (2007). Jasmonates: an update on biosynthesis, signal transduction and action in plant stress response, growth and development. Ann. Bot. 100, 681–697. 10.1093/aob/mcm079 17513307 PMC2749622

[B56] Winter D. Vinegar B. Nahal H. Ammar R. Wilson G. V. Provart N. J. (2007). An 'electronic fluorescent pictograph' browser for exploring and analyzing large-scale biological data sets. PLoS ONE 2:e718. 10.1371/journal.pone.0000718 17684564 PMC1934936

[B57] Yu G. H. Zou J. Feng J. Peng X. B. Wu J. Y. Wu Y. L.. (2014). Exogenous gamma-aminobutyric acid (GABA) affects pollen tube growth via modulating putative Ca^2+^-permeable membrane channels and is coupled to negative regulation on glutamate decarboxylase. J. Exp. Bot. 65, 3235–3248. 10.1093/jxb/eru171 24799560 PMC4071839

[B58] Zik M. Arazi T. Snedden W. A. Fromm H. (1998). Two isoforms of glutamate decarboxylase in *Arabidopsis* are regulated by calcium/calmodulin and differ in organ distribution. *Plant Mol*. Biol. 37, 967–975. 10.1023/A:1006047623263 9700069

