# Supplementary material for: Insect Herbivory-Elicited GABA Accumulation in Plants is a Wound-Induced, Direct, Systemic, and Jasmonate-Independent Defense Response
Source: Front Plant Sci. 2015 Dec 22;6:1128. doi: 10.3389/fpls.2015.01128 (PMC4686679; doi:10.3389/fpls.2015.01128)
Supplement: Supplementary file 1 [file DataSheet1.pdf]

Supplementary material

TABLE S1. List of primers used for RT-PCR

Gene	Primer sequence	Orientation	Function
GAD1	5'-CTTGAACGATCTCTTGGTCG-3'	Forward	RT-PCR
	5'-CGCTGTTAGATTCACTCTTCTC-3'	Reverse	RT-PCR
GAD2	5'-CTGTCTGCACCATGTTCGG-3'	Forward	RT-PCR
	5'-CACACCATTCATCTTCTTCC-3'	Reverse	RT-PCR
GAD3	5'-GCACATTTTTCCCTTTACTTTTCTTTAGC-3'	Forward	RT-PCR
	5'-GCTACTAACGGAACGCCG-3'	Reverse	RT-PCR
GAD4	5'-CATTTCAAACCCAAAAATCAAAGTTCG-3'	Forward	RT-PCR
	5'-GCAAATTGTGTTCTTGTTGG-3'	Reverse	RT-PCR
GAD5	5'-GCAAGGTACTTTGAGGTAGAGC-3'	Forward	RT-PCR
	5'-CCAATACTTAGTGATATCCTCC-3'	Reverse	RT-PCR
GAD1	5'-GGCAAGTGGAGGATTCATTG-3'	Forward	qRT-PCR
	5'-TTTCTCCAGATCACCCAACC-3'	Reverse	qRT-PCR
GAD2	5'-GAGAAATTCGCTCGGTACTTCGAG-3'	Forward	qRT-PCR
	5'-GTGTTCTCGTCTACCATTTCTGCTG-3'	Reverse	qRT-PCR
GAD3	5'-CTTTAGGTGACGGTGAAGCCG-3'	Forward	qRT-PCR
	5'-TGGCTCCGGTTACAATATTAGGT-3'	Reverse	qRT-PCR
GAD4	5'-GCTGATTCGTCTTGGATTCG-3'	Forward	qRT-PCR
	5'-AAACGCCACTAACGGAACAC-3'	Reverse	qRT-PCR
GAD5	5'-CAGGATTGCACATCTTGCTG-3'	Forward	qRT-PCR
	5'-CCACAAGGCGTTTCCAATAC-3'	Reverse	qRT-PCR
RPS18	5'-GTCTCCAATGCCCTTGACAT-3'	Forward	qRT-PCR
	5'-TCTTTCCTCTGCGACCAGTT-3'	Reverse	qRT-PCR
VSP2	5'-ACGACTCCAAAACCGTGTGCAA-3'	Forward	qRT-PCR
	5'-CGGGTCGGTCTTCTCTGTTCCGT-3'	Reverse	qRT-PCR
AOS	5'-AAGCCACGCGGCGTTTA-3'	Forward	qRT-PCR
	5'-GGAGTCTCCGTCTCCGGTCCA-3'	Reverse	qRT-PCR
LOX2	5'-ACGCTCGTGCACGCCAAAGT-3'	Forward	qRT-PCR
	5'-CCTCAGCCAACCCCCTTTTGA-3'	Reverse	qRT-PCR
JAR1	5'-TCCGTTTCGTCTGATCGGGATGT-3'	Forward	qRT-PCR
	5'-AGCTTCTTCAGGGTCAGTAGCGT-3'	Reverse	qRT-PCR
JAZ10	5'-TCGAGAAGCGCAAGGAGAGATTAGT-3'	Forward	qRT-PCR
	AGCAACGACGAAGAAGGCTTCAA-3'	Reverse	qRT-PCR

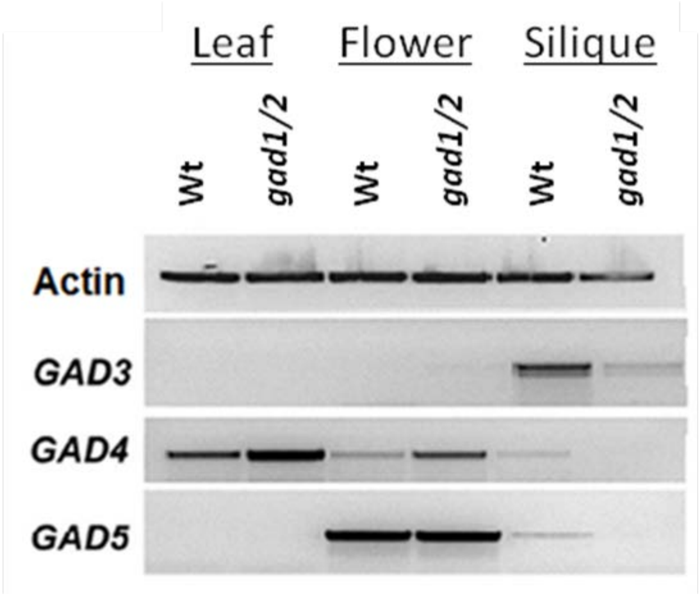

FIGURE S1. ***GAD3-5*** **expression in different Arabidopsis tissues.**

Leaves, flowers and young siliques of wild-type and *gad1/2* plants were investigated, all grown under greenhouse conditions. Specific primers amplifying the respective genes were used.

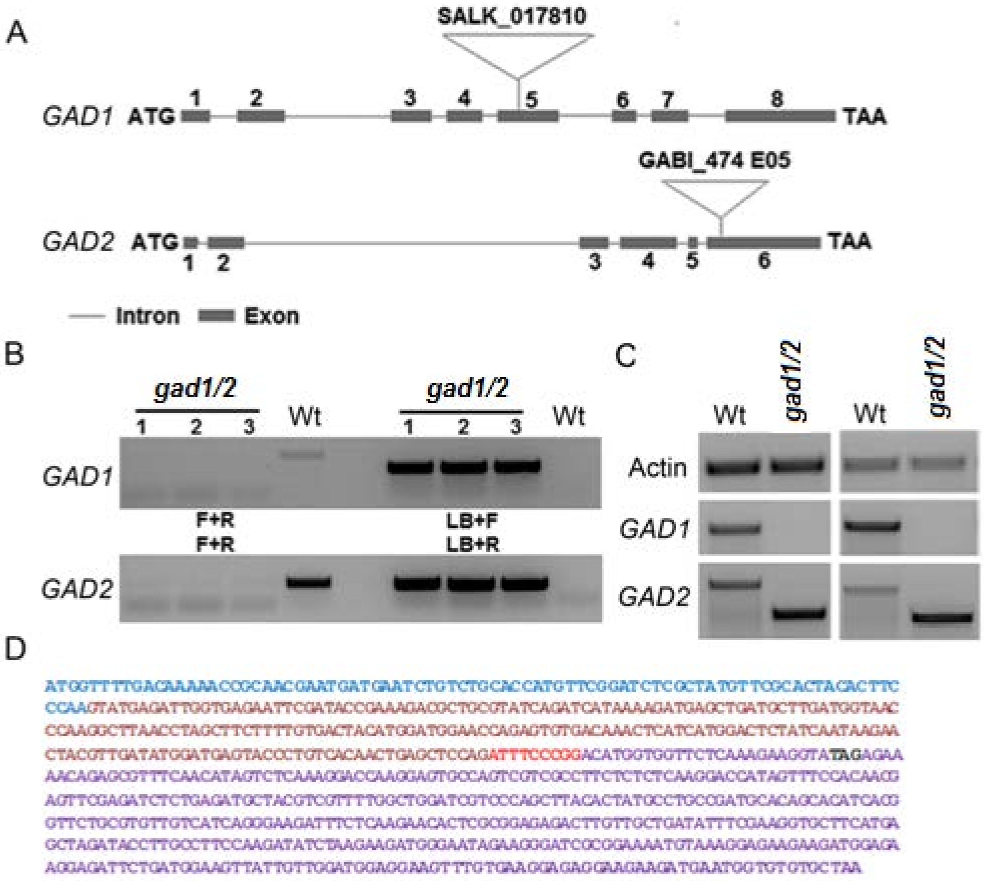

FIGURE S2. Molecular characterization of *gad1* and *gad2* T-DNA insertion mutants.

(**A**) Schematic representation of the T-DNA insertions in *GAD1* and *GAD2* genes. (**B**) Screening of *gad1* and *gad2* mutants with gene- and T-DNA-specific primer combinations. F, R and LB represent gene-specific forward, reverse and T-DNA-specific primers, respectively. (**C**) Transcript analysis of *GAD1* and *GAD2* genes from wild-type (Wt) and *gad1/2* plants in shoots (left) and roots (right). (**D**) Sequence of the truncated *GAD2* transcript. Sequences in blue, brown and purple represent exon 1, exon 2 and exon 6, respectively. Sequences in red are of unknown origin, probably intron, but inserted between exon 2 and exon 6. Sequences in bold black represent a stop codon due to a frame shift.

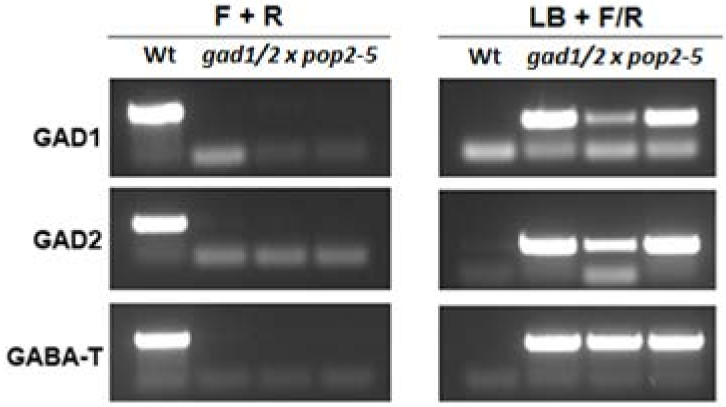

FIGURE S3. Genotyping of *gad1/2 x pop2-5* triple mutants.

For the analysis T-DNA- and gene-specific primers were used. F, R and LB represent gene-specific forward, reverse and T-DNA-specific primers, respectively.

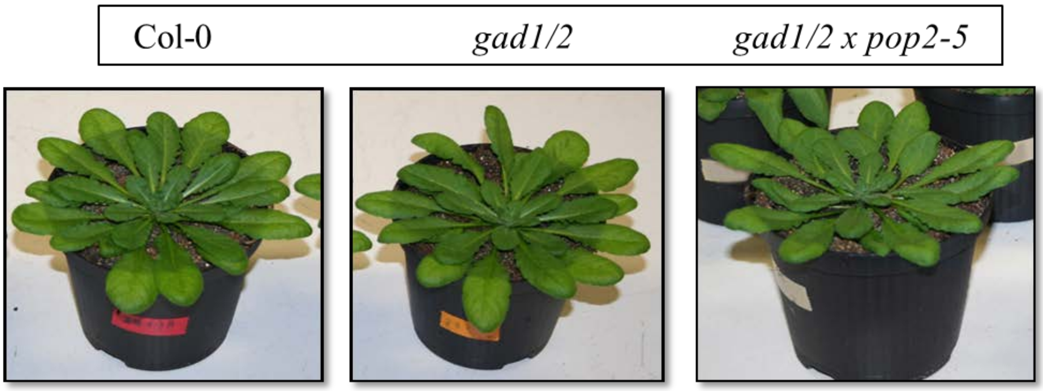

FIGURE S4. Phenotype of WT and GABA-mutant plants.

Phenotype of WT Col-0 (left), *gad1/2* (middle) and *gad1/2 x pop2-5* plants (right) after 4-5 weeks of growth. All plants for one experiment have the same germination date. The plants shown represent the typical phenotype observed in multiple replicates.

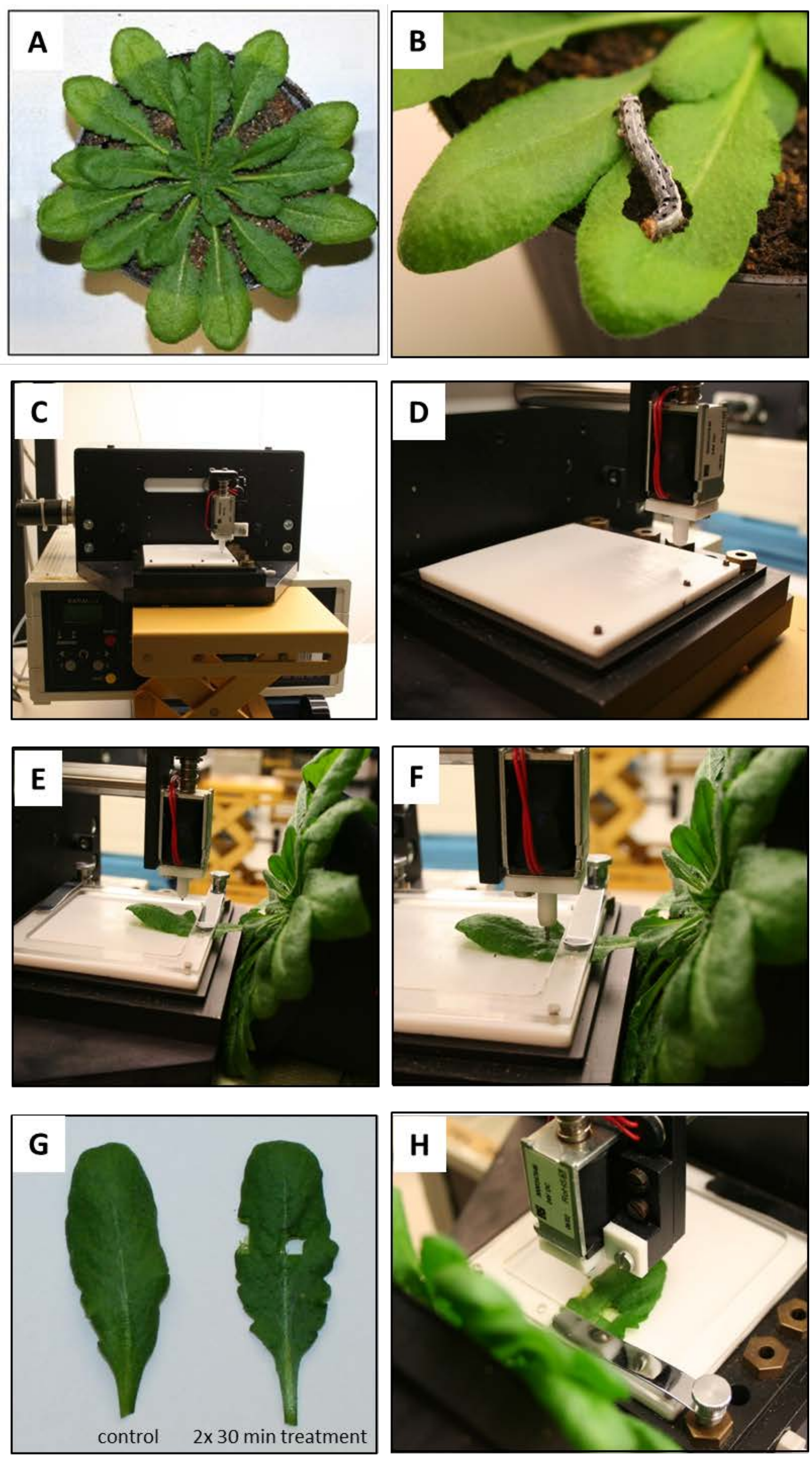

FIGURE S5. Herbivore and herbivory-related treatment of *Arabidopsis* plants. Shown are the size of 4-5 week old plants used for experiments (**A**), herbivore treatment (**B**) and treatment by mechanical wounding with MecWorm (**C-H**). For MecWorm treatment (**C**, **D**), 1 leaf of a potted plant was fixed in the machine (**E**) and punched every 5 s over a variable time period to mimic the larval feeding behavior (**F**). The wounded leaf (**G**, **H**) was collected for further analysis.

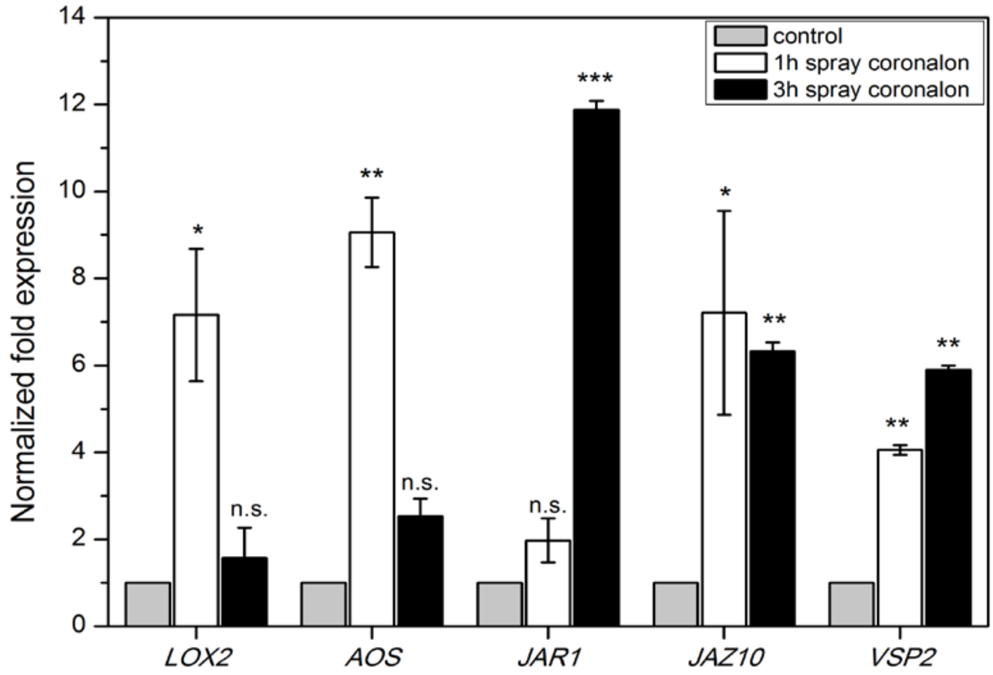

FIGURE S6. Induction of JA-biosynthesis and JA-responsive genes upon coronalon spray.

Normalized fold expression (± SE, n=6) of *LOX2*, *AOS*, *JAR1*, *JAZ10* and *VSP2* after 1 (white) and 3 h (black) of coronalon treatment. Plants were sprayed with 1 ml of 50 µM coronalon (50 nmol). Expression was normalized to the plant *RPS18* mRNA level. For control (grey), plants were sprayed with the same volume of water, its expression level was set to 1. Statistically significant differences between the control and the respective treatment (1 h / 3 h) was analyzed by t-test (for each gene separately), *P=<0.05, **P= <0.01, ***P = <0.001.
